# β-Cryptoxanthin Alleviates Diet-Induced Nonalcoholic Steatohepatitis by Suppressing Inflammatory Gene Expression in Mice

**DOI:** 10.1371/journal.pone.0098294

**Published:** 2014-05-23

**Authors:** Masuko Kobori, Yinhua Ni, Yumiko Takahashi, Natsumi Watanabe, Minoru Sugiura, Kazunori Ogawa, Mayumi Nagashimada, Shuichi Kaneko, Shigehiro Naito, Tsuguhito Ota

**Affiliations:** 1 National Food Research Institute, National Agriculture and Food Research Organization, Tsukuba, Ibaraki, Japan; 2 Department of Cell Metabolism and Nutrition, Brain/Liver Interface Medicine Research Center, Kanazawa University, Kanazawa, Ishikawa, Japan; 3 Department of Disease Control and Homeostasis, Kanazawa University Graduate School of Medical Science, Kanazawa, Ishikawa, Japan; 4 Citrus Research Division, NARO Institute of Fruit Tree Science, National Agriculture and Food Research Organization, Shimizu, Shizuoka, Japan; 5 Grape and Persimmon Research Division, NARO Institute of Fruit Tree Science, National Agriculture and Food Research Organization, Higashi-hiroshima, Hiroshima, Japan; State University of Rio de Janeiro, Biomedical Center, Institute of Biology, Brazil

## Abstract

Recent nutritional epidemiological surveys showed that serum β-cryptoxanthin inversely associates with the risks for insulin resistance and liver dysfunction. Consumption of β-cryptoxanthin possibly prevents nonalcoholic steatohepatitis (NASH), which is suggested to be caused by insulin resistance and oxidative stress from nonalcoholic fatty liver disease. To evaluate the effect of β-cryptoxanthin on diet-induced NASH, we fed a high-cholesterol and high-fat diet (CL diet) with or without 0.003% β-cryptoxanthin to C56BL/6J mice for 12 weeks. After feeding, β-cryptoxanthin attenuated fat accumulation, increases in Kupffer and activated stellate cells, and fibrosis in CL diet-induced NASH in the mice. Comprehensive gene expression analysis showed that although β-cryptoxanthin histochemically reduced steatosis, it was more effective in inhibiting inflammatory gene expression change in NASH. β-Cryptoxanthin reduced the alteration of expression of genes associated with cell death, inflammatory responses, infiltration and activation of macrophages and other leukocytes, quantity of T cells, and free radical scavenging. However, it showed little effect on the expression of genes related to cholesterol and other lipid metabolism. The expression of markers of M1 and M2 macrophages, T helper cells, and cytotoxic T cells was significantly induced in NASH and reduced by β-cryptoxanthin. β-Cryptoxanthin suppressed the expression of lipopolysaccharide (LPS)-inducible and/or TNFα-inducible genes in NASH. Increased levels of the oxidative stress marker thiobarbituric acid reactive substances (TBARS) were reduced by β-cryptoxanthin in NASH. Thus, β-cryptoxanthin suppresses inflammation and the resulting fibrosis probably by primarily suppressing the increase and activation of macrophages and other immune cells. Reducing oxidative stress is likely to be a major mechanism of inflammation and injury suppression in the livers of mice with NASH.

## Introduction

Nonalcoholic fatty liver disease (NAFLD) is one of the most prevalent forms of chronic liver disease in the developed countries and is frequently associated with obesity, metabolic syndrome, and type 2 diabetes. Nonalcoholic steatohepatitis (NASH), an advanced form of NAFLD, is characterized by hepatocellular steatosis along with lobular inflammation and fibrosis and may lead to liver cirrhosis and hepatocellular carcinoma [1]. Although insulin resistance, increased oxidative stress and subsequent lipid peroxidation, and increased proinflammatory cytokine release are believed to be the major causes of progression to NASH, the mechanisms have not been fully elucidated [1], [2]. In addition, no prevention or treatment of NASH has been fully established. Dietary modification and gradual weight loss are current mainstays of NASH treatment. The lipophilic antioxidant vitamin E has been studied as a candidate for NASH treatment. Recently, Sanyal *et al.* showed that vitamin E is superior to placebo for NASH treatment in adults without diabetes in a multicenter, randomized, placebo-controlled, double-blind clinical trial [3]. Vitamin E was associated with reduction in hepatic steatosis and lobular inflammation but not fibrosis [3].

β-Cryptoxanthin is a xanthophyll carotenoid that is routinely found in human plasma. Similar to other carotenoids, it shows antioxidant action [4], [5]. Serum β-cryptoxanthin concentrations were inversely associated with indices of oxidative DNA damage and lipid peroxidation [6]. Serum carotenoid concentrations were correlated with intake of fruits and vegetables [7]–[9].

Since β-cryptoxanthin is especially found in Satsuma mandarin (*Citrus unshiu* Marc.), its serum concentration reflects the amount of Satsuma mandarin intake among the residents of an area in which the mandarin is considerably more popular than in the rest of Japan [9], [10]. Further epidemiological studies have shown that serum β-cryptoxanthin concentrations are inversely associated with homeostasis model assessment-insulin resistance and alcohol-induced increase in serum γ-glutamyltransferase in nondiabetic subjects and alcohol drinkers, respectively [11], [12]. Thus, β-cryptoxanthin may prevent or alleviate NASH by suppressing oxidative stress or insulin resistance. In this report, we present the first evidence that β-cryptoxanthin suppressed induction of inflammatory gene expression and alleviated NASH in mice fed a high-cholesterol and high-fat (CL) diet.

## Material and Methods

### Purification of β-cryptoxanthin

Nonesterified β-cryptoxanthin for experiments was prepared from the raw centrifuged pulp of Satsuma mandarin juice processed as described [13]. In brief, the raw centrifuged pulp was subjected to enzymatic degradation, and then a precipitate was recovered following tubular centrifugation. The water contained in the precipitate was replaced with acetone. β-Cryptoxanthin was extracted from the acetone-substituted precipitate with hexane. After hexane removal, the extract was sequentially separated into soluble and insoluble portions with hexane, acetone, ethanol, and a hexane/ethanol (3∶7) mixture at −30°C using a centrifuge. β-Cryptoxanthin was concentrated in the hexane-soluble, acetone-soluble, ethanol-insoluble, and hexane/ethanol-insoluble portions in each treatment. The concentrated hexane/ethanol-insoluble portion was dissolved in hexane/ethanol (1∶1) and hydrolyzed with 10% potassium hydroxide in ethanol overnight at room temperature. After the addition of water, the organic phase was recovered and concentrated. The concentrate was dispersed in hexane by sonication. The insoluble substance was filtered and recrystallized from ethanol to afford nonesterified β-cryptoxanthin by filtration. According to HPLC analysis, the purity of the β-cryptoxanthin obtained was 96%. The raw pulp (10.0 kg) produced 0.74 g of β-cryptoxanthin (yield, 40%).

### Ethic statement

All animal procedures were performed in accordance with the standards set forth in the Guidelines for the Care and Use of Laboratory Animals at the Takara-Machi campus of Kanazawa University, Japan. The protocol was approved by the Committee on the Ethics of Animal Experiments of Kanazawa University (Approved number: AP-132887). The mice were housed in colony cages with a 12-h light/12-h dark cycle and allowed food and water *at libitum*. All surgeries were performed under sodium pentobarbital anesthesia, and all efforts were made to minimize suffering.

### Treatments

Eight-week-old male C57BL/6J mice were divided into three groups: (1) control mice fed a standard chow (CRF-1, Charles River Laboratories, Yokohama, Japan), (2) mice fed a CL diet containing 38.23% CRF-1, 60% cocoa butter, 1.25% cholesterol, and 0.5% sodium cholate, and (3) mice fed a CL diet containing 0.003% β-cryptoxanthin [14].

### Histological Examination and Immunohistochemistry

After 12 weeks of feeding, the mice were killed by cervical dislocation under ether anesthesia. The livers were immediately removed and weighed. A large portion of each liver was snap-frozen in liquid nitrogen for subsequent RNA studies. The remaining tissue was fixed in 10% buffered formalin, processed, and embedded in paraffin for hematoxylin–eosin (H&E) staining, Azan staining, and Sirius red staining. For immunohistochemical analysis, tissue slides were immunostained with mouse monoclonal anti-human α-smooth muscle actin (α-SMA; Dako Japan, Kyoto, Japan), or anti-mouse F4/80 (Abcam, Cambridge, UK) antibodies. This was followed by immunoperoxidase staining using the Envision kit (Dako Japan). Histological examination was performed for 4 mice in each group. Macrophage was quantitated by calculating the F4/80-positive area in 30 fields of 3 slides for each individual mouse using 5 mice for each group as described previously [15].

### Determination of Liver Lipid Levels

Total liver lipids were extracted using a modification of the method of Folch and Lees [16]. Briefly, snap-frozen liver tissues (150 mg) were homogenized and extracted twice with the chloroform: methanol (2∶1 v/v) solution. The organic layer was dried under nitrogen gas and resolubilized in chloroform. An aliquot was resuspended in an aqueous solution containing 2% Triton X-100 for the determination of triglyceride (TG) and total cholesterol (TC) mass. TG and TC levels were determined using a commercial colorimetric method (Wako Pure Chemicals Industries, Osaka, Japan). The concentration of thiobarbituric acid reactive substances (TBARS) was determined using a commercial colorimetric method (Cayman Chemical Company, Ann Arbor, MI, USA).

### DNA microarray analysis

Total RNA was isolated from the frozen liver using the GenElute Mammalian Total RNA Miniprep kit (Sigma-Aldrich Japan, Tokyo, Japan). Fragmented biotin-labeled aRNA was synthesized from the total RNA of each mouse using the GeneChip 3′ IVT Express Kit (Affymetrix Japan KK, Tokyo, Japan) and then hybridized to a GeneChip Mouse Genome 430 2.0 array (Affymetrix) at 45°C for 16 h. After hybridization, the probe array was washed and stained using GeneChip Fluidics Station 450 (Affymetrix) and then scanned (GeneChip Scanner 3000; Affymetrix) using GeneChip Operation Software Ver. 1.4 (Affymetrix). The data discussed in this publication have been deposited in NCBI's Gene Expression Omnibus [17] and are accessible through GEO Series accession number GSE51432 (http://www.ncbi.nlm.nih.gov/geo/query/acc.cgi?acc=GSE51432).

Data analysis was performed using Microarray Suite 5.0 (MAS5, Affymetrix) and GeneSpring Ver. 11.5 (Agilent Technologies, Santa Clara, CA, USA). The raw data collected from the GeneChip Operating System (Affymetrix) were uploaded onto GeneSpring Ver. 11.5 (Agilent Technologies) for further analysis. The gene expression data from each chip were then normalized to the 75th percentile of all measurements and from each probe were normalized to the median expression of matched intensity of control samples from mice fed the CRF-1 diet for that gene across all samples. We then filtered out the probe sets that were not called Present by the MAS5 detection call in at least 100% of the samples in 1 treatment group. Statistical analysis of differences in gene expression levels among the groups of mice fed the control, CL, and CL diets containing β-cryptoxanthin was performed using Welch's one-way ANOVA followed by the Tukey's post hoc test with Benjamin–Hochberg multiple corrections. The corrected p values of <0.05 were considered significant.

Hepatic genes that were significantly up- or downregulated in diet-induced NASH and genes whose expression levels were significantly altered by β-cryptoxanthin, leading to levels closer to the control levels, were analyzed using Ingenuity Pathway Analysis (Ingenuity Systems, www.ingenuity.com). This analysis identified biological functions that were most significant to the data set. A right-tailed Fisher's exact test was used to calculate a *p* value denoting the probability that each biological function for that data set was due to its change alone. An activation *z*-score was calculated as a measure of activation of biological function and functional or translational activation of upstream regulators. An absolute *z*-score of below (inhibited) or above (activated) 2 was considered significant.

Customized DNA microarray analysis was performed as described [18]–[20]. DNA oligonucleotide probes were synthesized for the detection of genes associated with NASH development and installed onto the fibrous DNA microarray Genopal (Mitsubishi Rayon, Tokyo, Japan), which comprises hollow plastic fibers containing a gel to which the probes can attach. Total RNA was amplified using the MessageAmpII biotin-enhanced amplification kit (Life Technologies, Carlsbad, CA, USA), according to the manufacturer's instructions and column purified. Biotinylated aRNA was fragmented using fragmentation reagents (Life Technologies) and then incubated at 95°C for 7.5 min. Hybridization, washing, and fluorescent labeling was performed using the Genopal UE-104 system (Mitsubishi Rayon). Hybridization was performed using a DNA microarray (Genopal) in hybridization buffer, 0.12 M Tris-HCl/0.12 M NaCl/0.05% Tween 20, and the fragmented biotinylated aRNA at 65°C for 16 h. The hybridized DNA microarray was washed in 1 mL of 0.12 M Tris-HCl/0.12 M NaCl/0.05% Tween 20 at 65°C and in 1 mL of 0.12 M Tris-HCl/0.12 M NaCl. The microarray was then fluorescently labeled with streptavidin-Cy5 (GE Healthcare Japan, Tokyo, Japan) and washed in 0.12 M Tris-HCl/0.12 M NaCl/0.05% Tween 20 at room temperature and in 1 mL of 0.12 M Tris-HCl/0.12 M NaCl. Hybridization signals were acquired using a DNA microarray reader and multibeam excitation technology (Yokogawa Electric Co., Tokyo, Japan). The DNA microarrays were scanned at multiple exposure durations of 0.1, 0.4, 1.0, 4.0, and 30 s. Intensity values with the optimal exposure condition for each spot were selected according to saturation. Statistical analyses were performed using GeneSpring Ver. 11.5 (Agilent Technologies). Data are expressed as the arithmetic mean ± standard error of the mean (SEM). The significance of differences between groups was determined by Welch's one-way ANOVA followed by the Tukey's post hoc test with Benjamin–Hochberg multiple corrections. The corrected p values of <0.05 were considered significant.

### Quantitative Real-Time PCR

Total RNA was isolated from the frozen liver using the GenElute Mammalian Total RNA Miniprep kit (Sigma-Aldrich Japan, Tokyo, Japan). cDNA was synthesized using the High Capacity cDNA Reverse Transcription Kit (Life Technologies). Quantitative real-time PCR was performed on CFX384 (Bio-Rad Laboratories, Hercules, CA, USA) using SYBR Green Master Mix (Takara Bio, Shiga, Japan) with appropriate primers as described [15], [21], [22]. The following primers were used for the reaction: CD3 forward (GACTATGAGCCCATCCGCAA), reverse (AACAAGGAGTAGCAGGGTGC); CD4 forward (TGTCACTCAAGGGAAGACGC), reverse (CGAAGGCGAACCTCCTCTAA); and CD8 forward (ACGAAGCTGACTGTGGTTGAT), reverse (AAAAGTAGACGGCCACTCCG).

### Statistical analysis

Data are expressed as the arithmetic mean ± SEM. The significance of differences between three groups was determined by Welch's one-way ANOVA followed by the Tukey's post hoc test using the statistical software SAS 9.4 (SAS Institute Japan Ltd., Tokyo) (GLM procedure with Welch option), the freeware R 3.0.2 (http://www.r-project.org/, qtukey function), and Excel 2013 (Microsoft, Tokyo). The two-group comparisons were analyzed by Student's t-test using GraphPad Prism 5 for Windows Ver. 5.01 (GraphPad Software, San Diego, CA). The p values of <0.05 were considered significant.

## Results

### β-Cryptoxanthin alleviates diet-induced NASH

**Figure 1:**
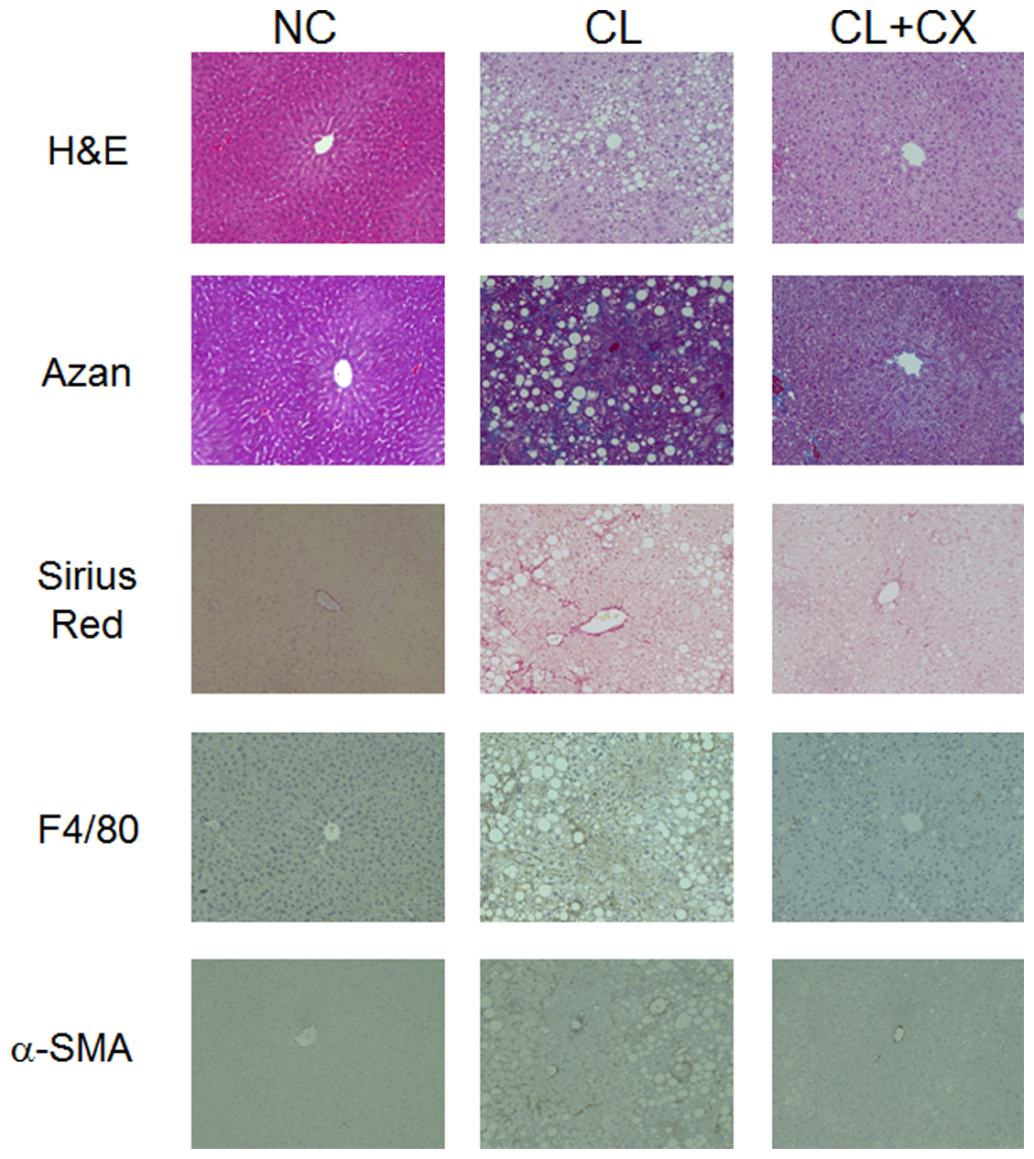
Representative liver histology of mice fed different diets. C57BL/6J mice were fed a standard chow (NC), a CL diet (CL), or a CL diet containing 0.003% β-cryptoxanthin (CL+CX) for 12 weeks. Liver sections were stained with H&E, Azan, Sirius red, anti-F4/80 antibody, and anti-α-SMA antibody. Original magnification is 200×, and scale bars  = 100 µm.

**Figure 2:**
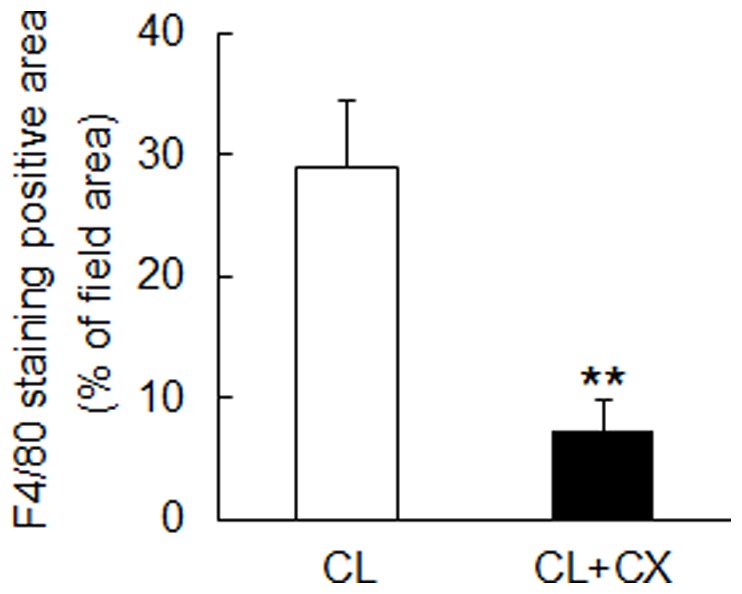
β-Cryptoxanthin reduced F4/80-positive hepatic macrophages. Liver sections of mice fed the CL diet (CL) or the CL diet containing 0.003% β-cryptoxanthin (CL+CX) for 12 weeks were stained with anti-F4/80 antibody. The F4/80-positive area was calculated in 30 fields of 3 slides for each individual mouse using 5 mice for each group as described previously [15]. **p<0.01, Student's t-test.

A CL diet induces a liver pathology similar to that of human NASH in C57BL/6J mice [14]. To elucidate the effect of β-cryptoxanthin on diet-induced NASH, we fed the C57BL/6J mice with a standard chow, CL diet, or CL diet containing 0.003% β-cryptoxanthin for 12 weeks. As shown in Table 1, the CL diet and β-cryptoxanthin did not affect body weight and energy intake in mice after 12 weeks of feeding. However, the CL diet significantly increased liver weight and TG and TC levels. β-Cryptoxanthin significantly reduced liver TG and TC levels after 12 weeks of feeding. The antioxidant β-cryptoxanthin significantly reduced increased levels of the marker of lipid peroxidation TBARS in mice fed the CL diet. Figure 1 shows representative photomicrographs of liver sections stained with H&E, Azan, Sirius red, anti-F4/80 antibody, and anti-α-SMA antibody. Lipid droplets accumulated in the hepatocytes of mice fed the CL diet for 12 weeks. Azan and Sirius red staining showed distinct collagen deposition, which is a characteristic of fibrosis, in the livers of mice fed the CL diet. The CL diet increased α-SMA-positive activated hepatic stellate cells and F4/80-positive hepatic macrophage Kupffer cells. Calculation of the F4/80-positive area is shown in Figure 2. β-Cryptoxanthin suppressed the accumulation of lipid droplets, collagen deposition, and F4/80-positive macrophages, which causes inflammation, in the livers of mice fed the CL diet. Thus, β-cryptoxanthin alleviated CL diet-induced steatosis, inflammation, and fibrosis in NASH in the mice.

**Table 1:** Metabolic parameters of mice fed with different diets for 12 weeks.

	NC	CL	CL+CX
Body weight (g)	31.7±1.4	32.6±0.8	32.9±0.6
Food intake (kcal/day/kg BW)	358.4±25.7	369.2±15.3	381.9±13.0
Liver weight/BW (%)	3.85±0.13^a^	4.63±0.12^b^	4.62±0.11^b^
Hepatic TG (mg/mg protein)	0.23±0.03^a^	0.66±0.05^b^	0.43±0.05^c^
Hepatic TC (mg/mg protein)	0.025±0.002^a^	0.248±0.016^b^	0.169±0.018^c^
Hepatic TBARS (nmol/mg protein)	0.16±0.01^a^	0.34±0.04^b^	0.18±0.04^a^

Data are expressed as the arithmetic mean ± SEM (*n* = 4 (NC) or n = 5 (CL and CL+CX)). Different superscripts indicate significant differences (p<0.05) between the groups.

### Comprehensive gene expression analysis using DNA microarrays

**Figure 3:**
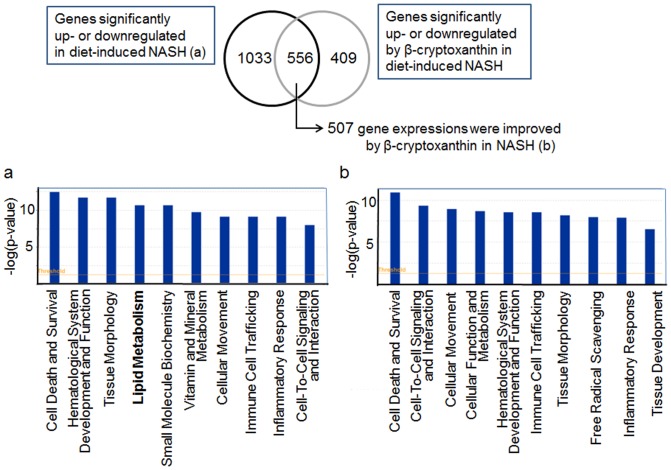
Top 10 biological functions of hepatic genes significantly improved by β-cryptoxanthin in diet-induced NASH in mice. (a)Top 10 biological functions of hepatic genes significantly up- or downregulated in NASH (b) Top 10 biological functions of hepatic genes significantly improved by β-cryptoxanthin, leading to levels closer to those of the control levels, in NASH. The functions that were most significant in the data set were identified by Ingenuity Pathway Analysis.

Comprehensive gene expression analysis using a DNA microarray showed that 2391 genes were differentially expressed in the livers of mice fed the standard chow, CL diet, and CL diet containing 0.003% β-cryptoxanthin after 12 weeks (*n* = 5, *p*<0.05 by one-way ANOVA). Among these genes, 1589 were significantly up- or downregulated in the livers showing CL diet-induced NASH compared with the normal livers of the control mice fed the standard chow (Figure 3(a)). Addition of β-cryptoxanthin to the CL diet altered the expression levels of 507 hepatic genes, leading to levels closer to those of the control mice (Figure 3(b)). The bar charts in Figure 2 show the top 10 biological functions putatively altered in NASH ((a), Table S1) and suppressed the alteration by β-cryptoxanthin ((b), Table S2). The expression of genes associated with liver injury (“cell death and survival”) and inflammation (“hematological system development and function,” “tissue morphology,” “cellular movement,” “immune cell trafficking and inflammatory responses,” and “cell-to-cell signaling and interaction”) was altered in NASH, and the alteration was suppressed by β-cryptoxanthin. The antioxidant β-cryptoxanthin suppressed the gene expression change associated with free radical scavenging (Figure 3(b), Table S2). β-Cryptoxanthin also suppressed the alteration of gene expression associated with cholesterol and other lipid metabolism (lipid metabolism, small-molecule biochemistry, and vitamin and mineral metabolism). The range of the *p*-value and the numbers of genes in the functions were 1.75×10^−6^–4.03×10^−2^ and 31 genes, 1.75×10^−6^–4.03×10^−2^ and 42 genes, and 3.72×10^−5^–3.78×10^−2^ and 22 genes, respectively. However, our result suggested that β-cryptoxanthin was more effecive in suppressing the alteration of gene expression associated with liver injury, inflammation, and free radical scavenging (Figure 3, Tables S1 and S2).

Table 2 shows the detailed biological functions that are predicted to be activated or suppressed by CL diet-induced NASH or β-cryptoxanthin. Many functions associated with accumulation, infiltration, and activation of macrophages, T lymphocytes, and other immune cells were predicted to be activated or suppressed in NASH. “Phagocytosis” and “cytotoxicity” were suggested to be induced in NASH. Functions associated with infiltration of immune cells, such as “chemotaxis,” “migration,” “homing,” and “trafficking,” were predicted to be activated in NASH. Inhibition of “morphology of leukocytes” reflects induction of the expression of genes related to signaling and activation of leukocytes. “Quantity of neutrophils” was predicted to be inhibited in NASH by the induction of expression of genes associated with quantity and recruitment of immune cells. β-Cryptoxanthin was suggested to suppress functions associated with “cell death” and “necrosis” in NASH. It was predicted to suppress the increase in T lymphocytes, activation of macrophages, cytotoxicity and migration of leukocytes, and inflammatory responses in NASH. The morphology of leukocytes was suggested to be preserved by β-cryptoxanthin. Production of reactive oxygen species was suggested to be increased in NASH and suppressed by β-cryptoxanthin.

**Table 2:** Predicted biological functions altered by high cholesterol diets and β-cryptoxanthin^a^.

Predicted biological functions altered by diet induced-NASH		Predicted biological functions improved by β-cryptoxanthin	
Functions Annotation	Activation State	Functions Annotation	Activation State
*Liver cell death*			
		cell death of liver cells (10 molecules)	↓
		necrosis of liver (14)	↓
*Accumulation, infiltration and activation of immune cells*			
accumulation of cells (33 molecules)	↑		
cell viability of mast cells (6)	↑		
proliferation of T lymphocytes (70)	↑		
quantity of T lymphocytes (69)	↑	quantity of T lymphocytes (27)	↓
		quantity of lymphatic system cells (9)	↓
		lack of T lymphocytes (15)	↑
quantity of neutrophils (30)	↓		
cytotoxicity of leukocytes (19), lymphocytes (16)	↑	cytotoxicity of leukocytes (11), lymphocytes (9)	↓
cytotoxicity of natural killer cells (10)	↑		
activation of cells (99), blood cells (94), macrophages (22)	↑	activation of cells (51), blood cells (48), macrophages (12)	↓
		activation of antigen presenting cells (21), leukocytes (45)	↓
inflammatory response (63)	↑	inflammatory response (31)	↓
phagocytosis (16)	↑		
phagocytosis of blood cells (19), cells (12), myeloid cells (9), phagocytes (7), by macrophages (4)	↑		
engulfment of cells (16)	↑		
engulfment of blood cells (13), myeloid cells (12), phagocytes (11), antigen presenting cells (8)	↑	engulfment of blood cells (6), myeloid cells (5), phagocytes (5), antigen presenting cells (5)	↓
		endocytosis (5)	↓
attachment of myeloid cells (4), phagocytes (4)	↑		
cell movement (109), leukocytes (91), phagocytes (71)	↑	cell movement (54), leukocytes (46), phagocytes (33)	↓
		cell movement of mononuclear leukocytes (18), lymphocytes (16)	↓
chemotaxis of leukocytes (45)	↑		
leukocyte migration (106)	↑	leukocyte migration (52)	↓
migration of cells (108)	↑	migration of cells (53)	↓
		migration of mononuclear leukocytes (15)	↓
		Lymphocyte migration (14)	↓
homing of leukocytes (47)	↑	homing of leukocytes (21)	↓
recruitment of leukocytes (40), phagocytes (31), mononuclear leukocytes (14)	↑		
transmigration of leukocytes (19)	↑		
trafficking of leukocytes (8)	↑		
extravasation of myeloid cells (7)	↑		
		cell spreading of phagocytes (6)	↓
		shape change of phagocytes (7)	↓
morphology of cells (74), leukocytes (61), mononuclear leukocytes (34), leukocytes (31), T lymphocytes (18)	↓	morphology of cells (32), leukocytes (27), mononuclear leukocytes (18), leukocytes (17), T lymphocytes (11)	↑
*Oxidative stress*			
production of reactive oxygen species (19)	↑	production of reactive oxygen species (13)	↓
*Lipid or carbohydrate metabolism*			
concentration of phosphatidic acid (5)	↓		
		quantity of carbohydrate (7)	↑

a The functions and canonical pathways that were most significant to the data set were identified by Ingenuity Pathway Analysis.

Upstream regulator analysis indicated the activation of nuclear receptors related to cholesterol metabolism along with cholesterol and cholic acid in CL diet-induced NASH (Table 3 (1)). Cytokines and molecules associated with immune response, inflammation, and proliferation were predicted to be activated in NASH. Among upstream regulators predicted to be inhibited in NASH, apolipoprotein E (*Apoe*), SREBF chaperone (*Scap*), and paraoxonase 1 (*Pon1*) are associated with cholesterol metabolism (Table 3 (2)). Other regulators related to proliferation and homeostasis were predicted to be inhibited in NASH (Table 3 (2)). β-Cryptoxanthin is predicted to inhibit the proinflammatory cytokine TNF (TNFα) and four other molecules associated with inflammation (Table 4).

**Table 3:** Upstream regulators predicted to be activated (a) or inhibited (b) in NASH^a^.

Molecule Type	Upstream Regulator
**(1)**	
chemical - endogenous	cholesterol (32 target molecules in dataset), cholic acid (14)
ligand-dependent nuclear receptor	Nr1h3 (16), Nr1h4 (14)
cytokine	Ifng (49), Ifnb1(25), Tnf (23), Il1b (20), Csf2 (12), Il5 (19), Il1a (4)
transcription regulator	Stat1 (16), Myc (19), Irf3 (6), Spi1 (7)
transmembrane receptor	Tlr4 (27)
peptidase	Plau (9)
enzyme	Cd38 (25)
kinase	Plk4 (8), Plk2 (8), Map3k7 (8), Tbk1 (10)
other	Myd88 (23), Ticam1 (19), Dock8 (13), Samsn1 (15), Arhgap21 (9), Sash1 (12), Tnfaip2 (4)
**(2)**	
transporter	Apoe (15 target molecules in dataset), Atp7b (12)
transcription regulator	Hnf4A (23), Smarcb1 (15)
phosphatase	Pon1 (4)
ligand-dependent nuclear receptor	Nr3C1 (15)
other	Scap (19), Socs1 (11)

a The functions and canonical pathways that were most significant to the data set were identified by Ingenuity Pathway Analysis.

**Table 4:** Upstream regulators predicted to be inhibited by β-cryptoxanthin in NASH^a^.

Upstream Regulator	Molecule Type	Activation z-score	p-value of overlap	Target molecules in dataset
Tnf	cytokine	−2.905	2.90E-04	Ccl4, Ccr5, Cd44, Gls, Hmox1, Ncf1, Ncf2, Ppara, Ppargc1a, Rxra, Tnfaip3, Vcam1
Spi1	transcription regulator	−2.219	3.75E-03	Cd14, Csf1r, Cybb, Emr1, Lyz1/Lyz2
Ticam1	other	−2.079	1.26E-02	Ccl4, Cd38, Cmpk2, Ifi204 (includes others), Pilra, Slc7a2, Tnfaip3, Vcam1
Myd88	other	−2.157	1.77E-02	Ccl4, Cd14, Cd38, Cmpk2,Inpp5d, Pilra, Slc7a2, Tnfaip3, Vcam1
Irf8	transcription regulator	−2.236	1.83E-02	Cebpa, Csf1r, Cxcl16, Emr1, Msr1

a The functions and canonical pathways that were most significant to the data set were identified by Ingenuity Pathway Analysis.

Table 5 shows the five canonical pathways that were most significant to the genes whose expression levels were altered by NASH or the genes whose expression levels were improved by β-cryptoxanthin. These top five canonical pathways, except for the “antigen presentation pathway” associated with macrophages, were associated with cholesterol synthesis or metabolism in the genes differentially expressed between control and NASH (Table 5 (1)). Pathways associated with macrophages, natural killer cells, and cytotoxic T cells were indicated as the most significant canonical pathways for genes whose expression levels were altered by β-cryptoxanthin, leading to levels closer to the control levels (Table 5 (2)).

**Table 5:** Top 5 canonical pathways of hepatic genes that were significantly altered by NASH (1) and improved by β-cryptoxanthin (2)^a^.

Ingenuity Canonical Pathways	p-value	Ratio
**(1)**		
LXR/RXR Activation	5.36E-07	28/113 (0.248)
LPS/IL-1 Mediated Inhibition of RXR Function	7.50E-07	40/193 (0.207)
Superpathway of Cholesterol Biosynthesis	7.81E-07	12/32 (0.375)
Antigen Presentation Pathway	1.61E-05	11/28 (0.393)
TR/RXR Activation	2.14E-05	20/79 (0.253)
**(2)**		
Fcγ Receptor-mediated Phagocytosis in Macrophages and Monocytes	7.54E-06	13/92 (0.141)
Natural Killer Cell Signaling	3.06E-05	12/92 (0.13)
CTLA4 Signaling in Cytotoxic T Lymphocytes	3.35E-05	11/79 (0.139)
TR/RXR Activation	3.79E-05	11/79 (0.139)
Production of Nitric Oxide and Reactive Oxygen Species in Macrophages	7.97E-05	16/166 (0.096)

a The functions and canonical pathways that were most significant to the data set were identified by Ingenuity Pathway Analysis.

### β-Cryptoxanthin suppressed gene expression associated with inflammation induced in NASH

**Figure 4:**
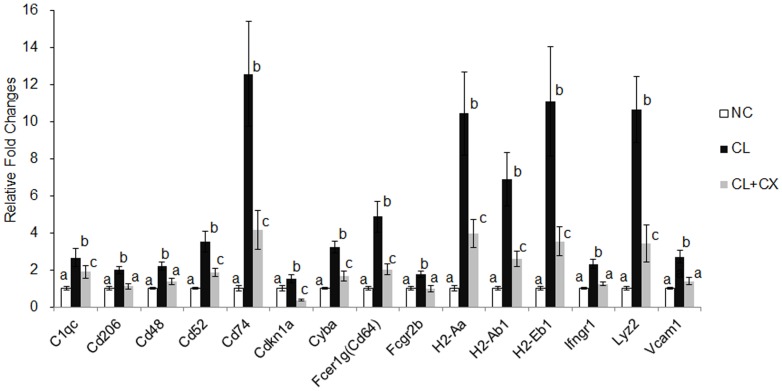
Hepatic gene expression levels significantly suppressed by β-cryptoxanthin in NASH. C57BL/6J mice were fed a standard chow (NC), a CL diet (CL), or a CL diet containing 0.003% β-cryptoxanthin (CL+CX) for 12 weeks. Hepatic gene expression was determined using a DNA microarray (Genopal, Mitsubishi Rayon). The degree of change (fold) was calculated compared with the livers of the control mice (NC). Data are expressed as the arithmetic mean ± SEM of 5 mice in each group. Different superscripts indicate significant differences (p<0.05) between the groups.

In our previous study, the fibrous DNA microarray efficiently detected the specific gene expression associated with lipid metabolism and inflammation with a high sensitivity [18], [20], [23]. To understand the effect of β-cryptoxanthin on the alteration of specific gene expression associated with cholesterol and other lipid metabolism, inflammation, and fibrosis, we then synthesized DNA oligonucleotide probes and prepared the fibrous DNA microarray carrying 174 genes (Table S3). The customized DNA microarray analysis showed that among the 24 genes significantly up- or downregulated by NASH, β-cryptoxanthin significantly suppressed 15 genes related to macrophages and other lymphocytes, and oxidative stress (Figure 4, Figure S1 and S2). Genes associated with MHC class II antigen presentation, *Cd74*, *H2-Aa*, *H2-Ab1*, and *H2-Eb1*, were strongly upregulated in NASH and downregulated by β-cryptoxanthin. β-Cryptoxanthin significantly suppressed the expression of the M2 macrophage markers *Cd206* in NASH. It also suppressed the expression of a constituent of the T cell receptor, *Fcer1g*. The expression of the TNFα-inducible genes *Cyba*, *Fcgr2b*, *Ifngr1*, and *Vcam1* was suppressed by β-cryptoxanthin in NASH. The expression of genes associated with cholesterol metabolism, including *Abca1*, *Ancg5*, and *Scd*, was significantly induced in NASH but not suppressed by β-cryptoxanthin (Figure S1).

### β-Cryptoxanthin suppressed the expression of T cell markers in NASH

**Figure 5:**
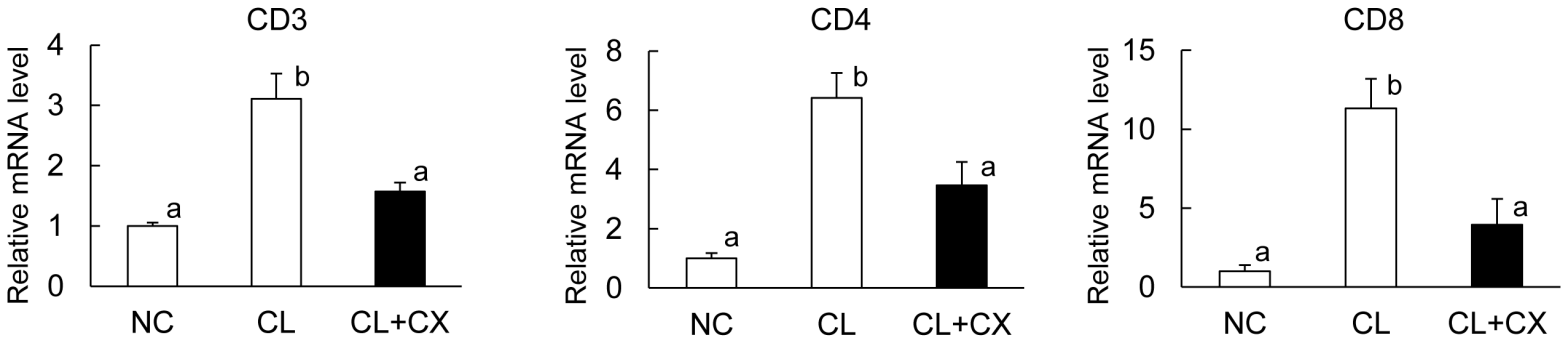
β-Cryptoxanthin reduced T cell accumulation. C57BL/6J mice were fed a standard chow (NC), a CL diet (CL), or a CL diet containing 0.003% β-cryptoxanthin (CL+CX) for 12 weeks. Hepatic expression levels of the T cell markers CD3, CD4, and CD8 were determined by quantitative RT-PCR analysis. Data are expressed as the arithmetic mean ± SEM (*n* = 4 (NC) or n = 5 (CL and CL+CX)). Different superscripts indicate significant differences (p<0.05) between the groups.

The results of Ingenuity Pathway Analysis suggested that β-cryptoxanthin suppressed the increase in T lymphocytes in NASH. The customized DNA microarray analysis indicated that the expression of T cell receptor was induced in NASH and suppressed by β-cryptoxanthin. We accordingly evaluated the expression of the cell surface antigens CD3, CD4, and CD8 by RT-PCR as markers of T cells, T helper cells, and cytotoxic T cells, respectively. The expression levels of CD3, CD4, and CD8 increased in CL diet-induced NASH (Figure 5). β-Cryptoxanthin significantly suppressed the CL diet-induced expression of CD3, CD4, and CD8 in the livers (Figure 5). Both helper and cytotoxic T cells were increased in NASH, and the increase was suppressed by β-cryptoxanthin. Thus, β-cryptoxanthin suppresses not only the accumulation of macrophages but also that of helper and cytotoxic T cells.

## Discussion

In the present study, we confirmed that the CL diet caused lipid accumulation mainly in the liver without affecting total body weight and food intake [14]. Importantly, β-cryptoxanthin reduced liver TG and TC levels without affecting either total body weight or caloric intake. This finding indicated that the effect of β-cryptoxanthin was not secondary to the reduction of caloric intake or body weight. β-Cryptoxanthin was shown to alleviate CL diet-induced steatosis, inflammation, and fibrosis in NASH in mice.

Comprehensive gene expression analysis showed that excessive intake of cholesterol, cholic acid, triglycerides, and fatty acids altered the expression of genes associated with cholesterol and other lipid metabolism in the liver. Cholesterol, cholic acid, the liver X receptor-α (LXR-α (*Nr1h3*)), which is activated by excess cholesterol and facilitates the transport of cholesterol out of cells [24], and the farnesoid X receptor-α (FXR-α (*Nr1h4*)), which is activated by excess cholic acid [25], were predicted to be activated upstream regulators. The regulators of cholesterol apolipoprotein E paeaoxonase 1, and SREBF chaperone were predicted to be inhibited upstream regulators. Most of the genes involved in “LXR/RXR activation” associated with cholesterol metabolism were upregulated and genes involved in the “superpathway of cholesterol biosynthesis” were downregulated in NASH. These results indicate that excess intake of cholesterol increased metabolism and suppressed synthesis. β-Cryptoxanthin partly suppressed the alteration of gene expression associated with cholesterol and other lipid metabolism. Oxidative stress is suggested to induce fat accumulation directly or indirectly through the exacerbation of insulin resistance [26]–[28]. Because β-cryptoxanthin reduced increased levels of the oxidative stress marker TBARS in NASH, it possibly suppressed fat accumulation by reducing oxidative stress in the liver.

Innate immune systems play a crucial role in NASH development [29]. Toll-like receptors (Tlrs), which are present on hepatocytes, Kupffer cells, and all other resident cells in the liver, are activated by saturated fatty acids or gut-derived endotoxins (LPS) and induced the expression of proinflammatory mediators in NASH. Excess fat induces hepatocyte c-jun N-terminal kinase and IκB kinase activation, which can induce hepatic insulin resistance, inflammatory cytokine expression, or cell death in the liver. The cytokines and chemokines further induce cell death, stimulate the production of reactive oxygen species, and recruit leukocytes from circulation [29]. Activation of Kupffer cells induces the production of Fas ligand and cytokines, such as TNFα, IL-1β and IL-6, inflammatory cell infiltrations, and subsequent liver injury and inflammation [29], [30]. Tosello-Trampont *et al.* reported that an increase in TNFα-producing Kupffer cells was crucial for triggering NASH via monocyte recruitment [30]. The adipocytokines TNFα and IL-6 derived from adipose tissues are strongly suggested to promote insulin resistance and inflammation in the liver [31]. β-Cryptoxanthin suppressed the accumulation of Kupffer cells in the livers of mice fed the CL diet. Comprehensive gene expression analysis suggested that whereas Tlr4, cytokines including TNFα, and some molecules regulated by LPS and/or TNFα were activated upstream regulators in NASH, TNFα and four other molecules regulated by LPS and/or TNFα were inhibited by β-cryptoxanthin. Moreover, β-Cryptoxanthin was suggested to reduce the quantity of T cells, activation of macrophages, and infiltration of leukocytes. Pathways associated with macrophages, monocytes, natural killer (NK) cells, and cytotoxic T cells were the most significant canonical pathways improved by β-cryptoxanthin. Thus, in addition to suppressing the accumulation of Kupffer cells, β-cryptoxanthin was suggested to suppress the signaling of TNFα and LPS, and activation and infiltration of macrophages, T cells, and NK cells. Oxidative stress is well recognized as a second hit to promote inflammation from steatosis. β-Cryptoxanthin probably suppresses both lipid accumulation and peroxidation as a potent antioxidant, thereby attenuating lipotoxicity-induced inflammation in NASH.

Comprehensive gene expression analysis suggested that antigen presentation and the following T cell activation were induced in NASH. Gene expression analysis using the customized fibrous DNA microarray confirmed that MHC-II antigens expressed on macrophages and other dendritic cells were strongly induced in NASH and significantly suppressed by β-cryptoxanthin to control levels. β-Cryptoxanthin suppressed the expression of the T cell receptor, which recognizes antigens bound to MHC-II molecules in NASH. CD4-positive T helper cells bind to MHC-II-positive cells, and CD8-positive cytotoxic T cells bind to MHC-I-positive cells. RT-PCR analysis showed that the expression of both T cell markers CD4 and CD8 was induced in NASH and reduced by β-cryptoxanthin. We also found that β-cryptoxanthin suppressed the CL diet-induced expression of the marker of M2 macrophages using the customized fibrous DNA microarray but not the comprehensive DNA microarray. Although M2 Kupffer cells were suggested to protect NAFLD by inducing M1 Kupffer cell apoptosis [32], β-cryptoxanthin suppressed the increase in both classical M1 and alternative M2 macrophages. β-Cryptoxanthin suppressed TNFα-inducible gene expression increased in NASH. The TNFα-inducible genes *Ifngr1*, and *Vcam1* have been reported to increase fibrosis in the liver [33]–[35]. Among genes whose expression levels were significantly reduced by β-cryptoxanthin in NASH, *Cd48* was reported to be upregulated in the livers of patients with NASH [36].

Mitochondrial free cholesterol accumulation was reported to sensitize hepatocytes to TNFα and Fas by inducing mitochondrial glutathione depletion [37]. The high-fat component of the CL diet was shown to accelerate oxidative stress induced by a high-cholesterol diet in the livers of CL57BL/6J mice [14]. Fat accumulation causes hepatocellular injury probably by increasing lipid peroxidation and metabolites of fatty acids [38]. Our results suggested that consumption of β-cryptoxanthin improved gene expression associated with “cell death and survival” and “free radical scavenging,” in particular, suppressing hepatic cell death and reducing the production of reactive oxygen species induced by the CL diet. β-Cryptoxanthin is likely to suppress oxidative stress and liver injury by scavenging reactive oxygen species and/or reducing their production. We previously reported that β-cryptoxanthin was highly accumulated in the liver of rats fed a diet containing the Satsuma mandarin extract for 8 weeks [39]. β-Cryptoxanthin probably accumulated in the liver and directly reduced the increased level of the oxidative stress marker TBARS in NASH.

This study includes the results of one-way ANOVA with unequal small sample sizes (n_1_ = 4, n_2_ = 5, and n_3_ = 5; Table 1 and Fig. 5). ANOVA is a parametric test under preconditions such as normality and homogeneity of variance, however, it is robust against the preconditions as long as all sample sizes are equal or nearly equal [40]. Nevertheless, we performed Welch's ANOVA followed by Tukey's post hoc test. Welch's ANOVA performs rather well when population variances are unequal [40]. Furthermore, we confirmed that significant differences (p<0.05) between treatments (Table 1 and Fig. 5) determined by Welch's ANOVA followed by Tukey's post hoc test were identical to those determined by the bootstrap and permutation-based multiple comparisons using the MULTTEST procedure (SAS Institute Japan Ltd., Tokyo), specifically designed for handling non-normal data [41]. The resource equation method for sample size determination is useful for small and complex biological experiments that involve several treatment groups for which the results are to be analyzed using ANOVA [42]. The experiment should be of an appropriate size if the error degrees of freedom (DF) in an ANOVA are somewhere between 10 and 20 [42]. In Table 1 and Fig. 5, DF = 11 for the error term in one-way ANOVA. Thus we confirmed the reliability of the results of statistical analysis in Table 1 and Fig. 5.

## Conclusions

Intake of the dietary xanthophyll β-cryptoxanthin reduced NASH development induced by a CL diet in mice. Gene expression analysis showed that although β-cryptoxanthin histochemically reduced steatosis, it was more effective in inhibiting inflammatory gene expression change in NASH. In fact, β-Cryptoxanthin reduced the induction of markers of macrophages, T helper cells, and cytotoxic T cells. The expression of LPS-inducible and/or TNFα-inducible genes was suppressed by β-cryptoxanthin, probably via the inhibition of macrophage activation. Thus, β-cryptoxanthin suppresses inflammation and subsequent fibrosis primarily by suppressing the increase and activation of macrophages and other immune cells. Reduction of oxidative stress is likely to be a major mechanism of suppression of inflammation and injury in the livers of mice with NASH. Our results provide an important clue to elucidate the molecular mechanism of alleviation effect of β-cryptoxanthin on CL diet-induced NASH.

## Supporting Information

Figure S1 **Hepatic gene expressions significantly altered in NASH.** C57BL/6J mice were fed a standard chow (NC), a CL diet (CL), or a CL diet containing 0.003% β-cryptoxanthin (CL+CX) for 12 weeks. Hepatic gene expression was determined using a DNA microarray (Genopal, Mitsubishi Rayon). The degree of change (fold) was calculated compared with the livers of the control mice (NC). Data are expressed as the arithmetic mean ± SEM of 5 mice in each group. Different superscripts indicate significant differences (p<0.05) between the three groups.
(TIF)

Figure S2 **Hepatic gene expressions did not significantly alter in NASH.** C57BL/6J mice were fed a standard chow (NC), a CL diet (CL), or a CL diet containing 0.003% β-cryptoxanthin (CL+CX) for 12 weeks. Hepatic gene expression was determined using a DNA microarray (Genopal, Mitsubishi Rayon). The degree of change (fold) was calculated compared with the livers of the control mice (NC). Data are expressed as the arithmetic mean ± SEM of 5 mice in each group.
(TIF)

Table S1 **Top 10 Biological functions of hepatic genes that were significantly up- or down regulated in diet-induced nonalcoholic steatohepatitis in mice.** The functions and canonical pathways that were most significant to the data set were identified by Ingenuity Pathway Analysis (Ingenuity Systems).
(PDF)

Table S2 **Top 10 Biological functions of hepatic genes that were significantly improved by β-cryptoxanthin in mice.** The functions and canonical pathways that were most significant to the data set were identified by Ingenuity Pathway Analysis (Ingenuity Systems).
(PDF)

Table S3 **DNA oligonucleotide probes for customized fibrous DNA microarray.**
(XLS)
