# Supplementary figures and images for: β-Cryptoxanthin Alleviates Diet-Induced Nonalcoholic Steatohepatitis by Suppressing Inflammatory Gene Expression in Mice
Source: PLoS One. 2014 May 23;9(5):e98294. doi: 10.1371/journal.pone.0098294 (PMC4032271; doi:10.1371/journal.pone.0098294)

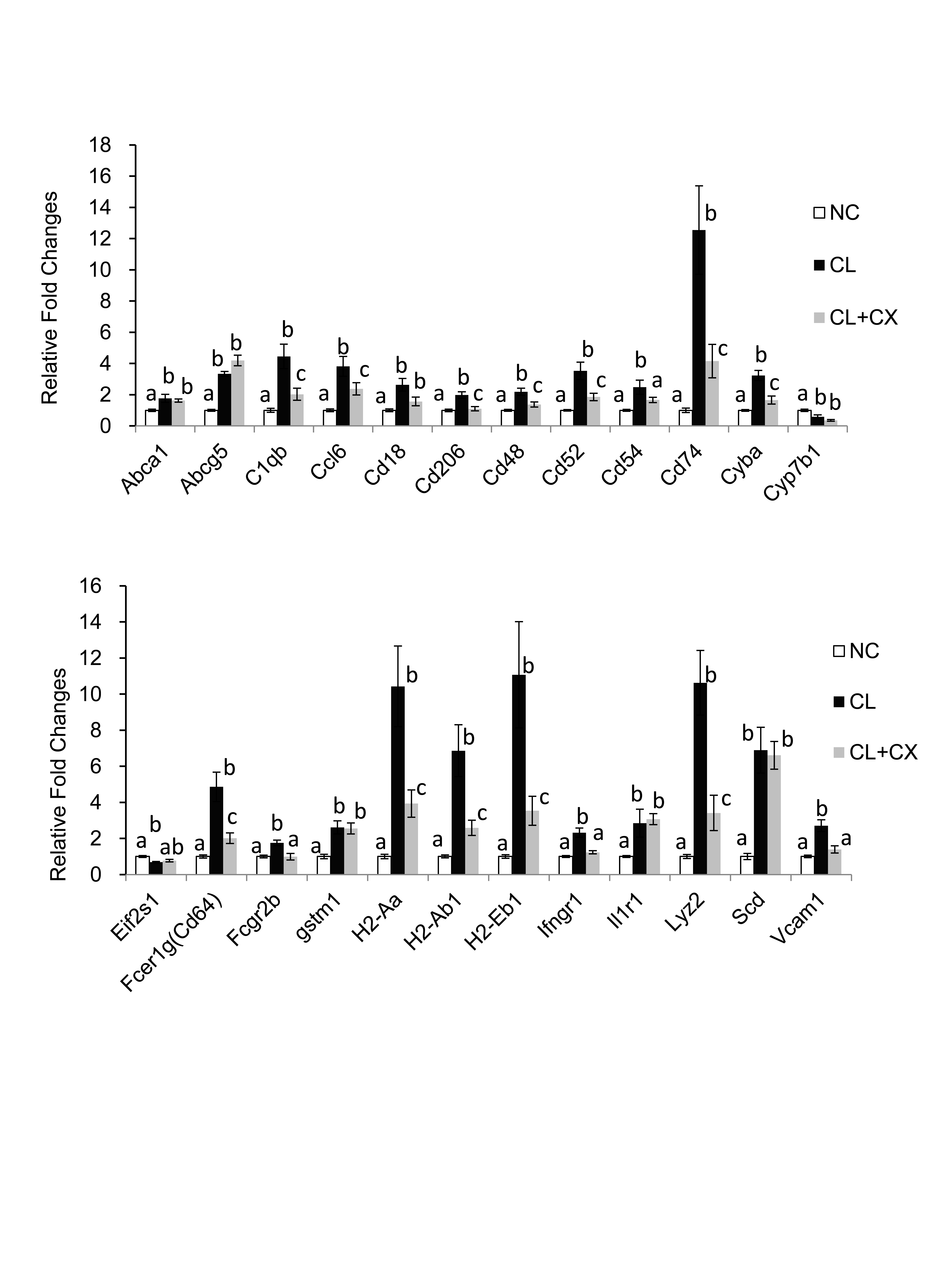

Supplement: Figure S1 — Hepatic gene expressions significantly altered in NASH. C57BL/6J mice were fed a standard chow (NC), a CL diet (CL), or a CL diet containing 0.003% β-cryptoxanthin (CL+CX) for 12 weeks. Hepatic gene expression was determined using a DNA microarray (Genopal, Mitsubishi Rayon). The degree of change (fold) was calculated compared with the livers of the control mice (NC). Data are expressed as the arithmetic mean ± SEM of 5 mice in each group. Different superscripts indicate significant differences (p<0.05) between the three groups. (TIF) [file pone.0098294.s001.tif]

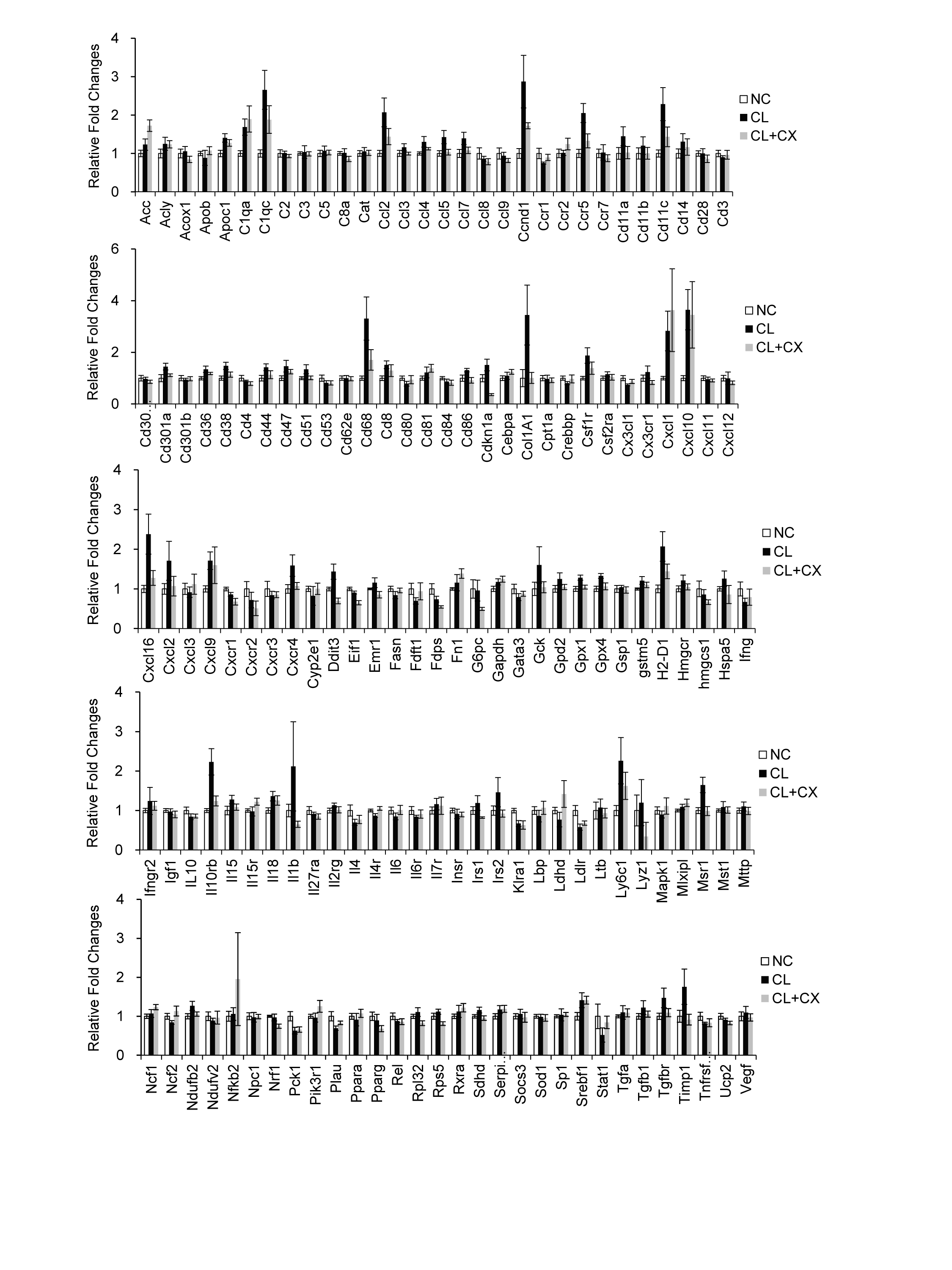

Supplement: Figure S2 — Hepatic gene expressions did not significantly alter in NASH. C57BL/6J mice were fed a standard chow (NC), a CL diet (CL), or a CL diet containing 0.003% β-cryptoxanthin (CL+CX) for 12 weeks. Hepatic gene expression was determined using a DNA microarray (Genopal, Mitsubishi Rayon). The degree of change (fold) was calculated compared with the livers of the control mice (NC). Data are expressed as the arithmetic mean ± SEM of 5 mice in each group. (TIF) [file pone.0098294.s002.tif]
