# Supplementary material for: β-Cryptoxanthin Alleviates Diet-Induced Nonalcoholic Steatohepatitis by Suppressing Inflammatory Gene Expression in Mice
Source: PLoS One. 2014 May 23;9(5):e98294. doi: 10.1371/journal.pone.0098294 (PMC4032271; doi:10.1371/journal.pone.0098294)
Supplement: Table S1 — Top 10 Biological functions of hepatic genes that were significantly up- or down regulated in diet-induced nonalcoholic steatohepatitis in mice. The functions and canonical pathways that were most significant to the data set were identified by Ingenuity Pathway Analysis (Ingenuity Systems). (PDF) [file pone.0098294.s003.pdf]

Biological Functions	p-value	No. of genes differentially expressed
**Cell death and Survival (49 functions)**	3.26E-13 - 3.92E-02	122
**Hematological System Development and Function (232 functions)**	1.86E-12 - 4.71E-02	256
**Tissue Morphology (31 functions)**	1.86E-12 - 4.49E-02	169
**Lipid Metabolism (56 functions)**	2.05E-11 - 4.71E-02	74
**Small Molecule Biochemistry (63 functions)**	2.05E-11 - 4.71E-02	99
**Vitamin and Mineral Metabolism (10 functions)**	1.76E-10 - 2.51E-02	47
**Cellular Movement (96 functions)**	7.26E-10 - 4.71E-02	112
**Immune Cell Trafficking (134 functions)**	7.26E-10 - 4.71E-02	161
**Inflammatory Response (122 functions)**	7.26E-10 - 4.71E-02	195
**Cell-To-Cell Signaling and Interaction (103 functions)**	9.95E-09 - 4.71E-02	148
