# Supplementary material for: β-Cryptoxanthin Alleviates Diet-Induced Nonalcoholic Steatohepatitis by Suppressing Inflammatory Gene Expression in Mice
Source: PLoS One. 2014 May 23;9(5):e98294. doi: 10.1371/journal.pone.0098294 (PMC4032271; doi:10.1371/journal.pone.0098294)
Supplement: Table S2 — Top 10 Biological functions of hepatic genes that were significantly improved by β-cryptoxanthin in mice. The functions and canonical pathways that were most significant to the data set were identified by Ingenuity Pathway Analysis (Ingenuity Systems). (PDF) [file pone.0098294.s004.pdf]

Biological Functions	p-value	No. of genes differentially expressed
**Cell death and Survival (50 functions)**	1,17E-11 – 4.59E-02	57
**Cell-To-Cell Signaling and Interaction (66 functions)**	4.39E-10 – 4.43E-02	74
**Cellular Movement (78 functions)**	1.10E-09 – 3.78E-02	56
**Cellular Function and Maintenance (58 functions)**	1.98E-09 – 4.82E-02	67
**Hematological System Development and Function (213 functions)**	2.70E-09 – 4.96E-02	113
**Immune Cell Trafficking (99 functions)**	2.70E-09 – 4.43E-02	77
**Tissue Morphology (33 functions)**	6.20E-09 – 4.91E-02	70
**Free Radical Scavenging (9 functions)**	1.02E-08 – 1.47E-02	16
**Inflammatory Response (109 functions)**	1.02E-08 – 4.43E-02	86
**Tissue Development (33 functions)**	2.83E-07 – 3.57E-02	36
